# Interaction between the Triglyceride Lipase ATGL and the Arf1 Activator GBF1

**DOI:** 10.1371/journal.pone.0021889

**Published:** 2011-07-18

**Authors:** Emy Njoh Ellong, Krishnakant G. Soni, Quynh-Trang Bui, Rachid Sougrat, Marie-Pierre Golinelli-Cohen, Catherine L. Jackson

**Affiliations:** 1 Laboratoire d'Enzymologie et Biochimie Structurales, Bat 34, Centre National de la Recherche Scientifique (CNRS), Gif-sur-Yvette, France; 2 Cell Biology and Metabolism Program, Eunice Kennedy Shriver National Institute of Child Health and Human Development, National Institutes of Health, Bethesda, Maryland, United States of America; Institut Pasteur, France

## Abstract

The Arf1 exchange factor GBF1 (Golgi Brefeldin A resistance factor 1) and its effector COPI are required for delivery of ATGL (adipose triglyceride lipase) to lipid droplets (LDs). Using yeast two hybrid, co-immunoprecipitation in mammalian cells and direct protein binding approaches, we report here that GBF1 and ATGL interact directly and in cells, through multiple contact sites on each protein. The C-terminal region of ATGL interacts with N-terminal domains of GBF1, including the catalytic Sec7 domain, but not with full-length GBF1 or its entire N-terminus. The N-terminal lipase domain of ATGL (called the patatin domain) interacts with two C-terminal domains of GBF1, HDS (Homology downstream of Sec7) 1 and HDS2. These two domains of GBF1 localize to lipid droplets when expressed alone in cells, but not to the Golgi, unlike the full-length GBF1 protein, which localizes to both. We suggest that interaction of GBF1 with ATGL may be involved in the membrane trafficking pathway mediated by GBF1, Arf1 and COPI that contributes to the localization of ATGL to lipid droplets.

## Introduction

Trafficking in the early secretory pathway requires two vesicular trafficking machineries, the Sec12-Sar1-COPII and GBF1-Arf1-COPI systems [1], [2]. GBF1 and its yeast homologues Gea1p and Gea2p are guanine nucleotide exchange factors (GEFs) for the small G protein Arf1 that specifically recruit the COPI coat to membranes to form COPI-coated vesicles [3], [4], [5]. GBF1 is a peripherally associated membrane protein that localizes to endoplasmic reticulum export site (ERES) membranes, promoting their maturation to the ER-Golgi intermediate compartment through recruitment of COPI and lipid modifying enzymes [6]. GBF1 also localizes to Golgi membranes, where it promotes formation of COPI vesicles that recycle material from the Golgi back to the ER [7]. Sec12 is an integral endoplasmic reticulum (ER) membrane protein that activates the Sar1 small G protein to promote COPII binding to membranes [6]. COPII recruitment aids in the formation of COPII-coated vesicles and creates membrane domains, ERES, competent for export of proteins from the ER, the first step in the secretory pathway [8]. GBF1 is a large multidomain protein that has several interacting partners, including the COPI coat complex and the membrane tether p115 that regulates membrane fusion [5], [9]. These proteins act downstream of Arf1 activation, suggesting that GBF1 acts as a scaffold to coordinate downstream events prior to its activation of Arf1. At the *trans*-Golgi Network and endosomes, two other Arf1 GEFs, Brefeldin A-Inhibited Guanine nucleotide exchange factor 1 (BIG1) and BIG2, function in recruitment of coats specific to these compartments [3], [4], [10].

Cells store energy in the form of triacylglycerol and cholesterol esters in structures known as lipid droplets (LDs) – also called lipid bodies, oil bodies and adiposomes [11], [12], [13], [14], [15]. These stores can be mobilized under conditions of nutrient deprivation through the action of lipases, including adipose triglyceride lipase (ATGL) and hormone sensitive lipase (HSL) [14], [16], [17]. ATGL, also known as PNPLA2 (for patatin-like phospholipase domain containing 2) is a member of a family of LD-associated lipases that catalyze the first step of triacylglycerol degradation upon stimulation of lipolysis [16], [18], [19]. The patatin domain, named after the plant lipase patatin, is found in a family of lipases that also includes cytosolic phospholipase A2 (cPLA2) [16], [18], [20]. The crystal structure of the patatin domain has been solved, revealing an active site unlike the typical alpha/beta hydrolase fold, and resembling that of cPLA2 [21]. CGI-58 is a well-studied interacting partner of ATGL, which binds directly to the patatin domain and acts as a co-activator which stimulates ATGL lipase activity [16], [22], [23], [24], [25]. ATGL and CGI-58, although highly upregulated in adipocytes, are present in non-adipocyte cells as well, and hence may play general roles in cellular lipid metabolism [17], [20], [26].

LDs have a central hydrophobic core in which triacylglycerol and cholesterol esters are stored, which is surrounded by a phospholipid monolayer. Lipid-droplet associated proteins coat the external surface of the LD, and include PAT (Perilipin/ADRP/TIP47) proteins and lipases such as ATGL and HSL [13], [15], [25], [27]. Recently, numerous studies have shown that LDs are not simply inert storage depots, but are dynamic organelles that interface with membrane trafficking pathways [11], [28], [29]. One of the first clues came from proteomics studies of purified LDs that turned up proteins involved in membrane trafficking including Rab and Arf family proteins and SNAREs [29], [30], [31], [32], [33]. Mulitple Rab proteins have been implicated in LD metabolism, with Rab18 and Rab5 having demonstrated roles in lipid droplet metabolism [34], [35]. Rab18 is localized to both the ER and LDs, and its trafficking to LDs is triggered by lipolytic stimulation, indicating a potential role in the breakdown of LDs during lipolysis [35]. The SNARE proteins SNAP23 and the Arf family member ARFRP1 have been shown to have a functional connection to LDs, and SNAP23 appears to play a direct role in LD fusion [36], [37].

We have shown recently that GBF1, Arf1 and COPI are required for delivery of lipid droplet-associated proteins such as the triglyceride lipase ATGL to LDs in mammalian cells [38], and a role for these proteins in LD morphology was discovered in genome-wide screens in *Drosophila melanogaster* [39], [40]. We also demonstrated a role for Sar1 and COPII in trafficking of ATGL to LDs [38]. We found that ATGL is tightly associated with membranes, and when its delivery to LDs is blocked, it accumulates on ER membranes [38]. However, some groups have reported localization of ATGL to the cytoplasm in addition to LDs [41], [42]. In cells depleted of GBF1, ATGL colocalizes with COPII components, at a subset of ERES [38]. These results support the conclusion that ATGL is tightly associated with the ER membrane from the time of its synthesis, and requires sorting by COPII and COPI for its trafficking to LDs. Although the COPII system is involved in targeting of ATGL to LDs, depletion of COPII components does not affect LD size, either in HeLa cells or in *Drosophila* S2 cells [38], [39], [40]. In contrast, depletion of GBF1 in HeLa cells or in *Drosophila* results in larger lipid droplets. In addition, a more severe ATGL delivery defect was seen when components of the GBF1-Arf1-COPI system were inactivated, compared to the COPII system. These results suggest a more direct role for GBF1, Arf1 and/or COPI in LD metabolism.

Our current study began with a yeast two-hybrid screen using the Sec7 domain of BIG2 as bait. We identified the C-terminal region of ATGL as an interacting partner, and this prompted us to investigate the potential connections between ATGL and the Arf1 GEFs. Surprisingly, we found little evidence of a physiological connection between ATGL and BIG2 or BIG1, but did find a role for GBF1, as described above. Here we show that several domains of GBF1 interact with ATGL directly, and that GBF1 and ATGL interact in mammalian cells.

## Results

### ATGL is an interacting partner of the Arf1 activator GBF1

**Figure 1:**
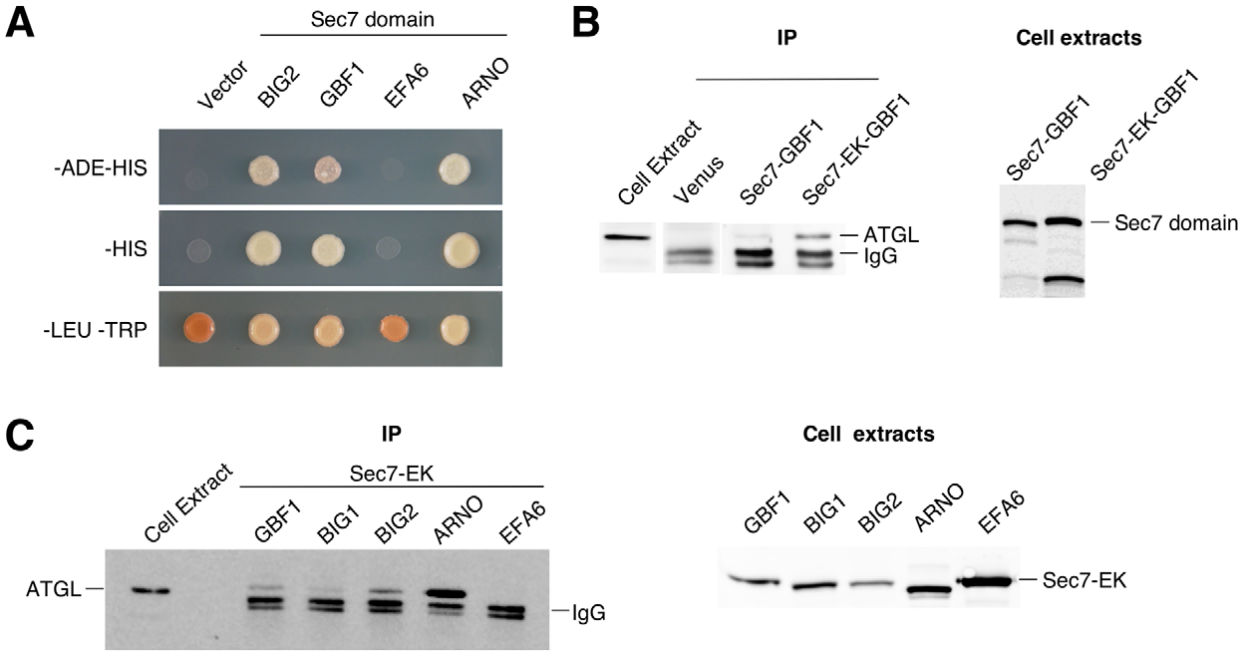
ATGL interacts with Arf1 GEF catalytic Sec7 domains. A- Yeast strain AH109 was transformed with the pACT2 library plasmid containing amino acids 367–504 of ATGL fused to the activation domain of Gal4p, and pGBKT7 vectors carrying the indicated Sec7 domain fused to the DNA-binding domain of Gal4p. Cells were spotted on the indicated selective plates and incubated for three days at 30°C. B-C- HA-tagged *H. sapiens* ATGL was coexpressed in Cos7 cells either with Venus alone, or with the indicated Venus-tagged Sec7 domain; Sec7-EK indicates a mutation of the catalytic glutamate residue to lysine. Immunoprecipitation was carried out with anti-GFP antibodies, and Cos7 cell lysates or eluted proteins after immunoprecipitation were analyzed by Western blotting using anti-HA antibody (left panels). Lysates from cells expressing the indicated Venus-GBF1 construct were analyzed by Western blotting using anti-GFP antibody (right panels). B- Wild type and E794K mutant Sec7 domains of GBF1. C- EK mutant Sec7 domains of GBF1, BIG1, BIG2, ARNO and EFA6.

We carried out a yeast two-hybrid screen with the Sec7 domain of the Arf1 GEF BIG2 as bait, screening a human brain cDNA library. After multiple rounds of selection, 132 positive clones were obtained and inserts sequenced. One clone contained the C-terminal portion (amino acids 367 to 504) of adipose triglyceride lipase (ATGL). We tested this clone for interaction with other Sec7 domains. We included those of GBF1 and ARNO, which have Arf1 as their preferred substrate, and EFA6, which is specific for Arf6. (Our BIG1 Sec7 domain constructs were not functional in yeast two-hybrid assays). The Arf1-specific GEFs GBF1 and ARNO showed a robust interaction with the C-terminus of ATGL, but no interaction was seen with the Sec7 domain of EFA6, whose preferred substrate is Arf6 (Figure 1A).

We co-expressed full-length ATGL and the Sec7 domains of GBF1, BIG1, BIG2 or EFA6 in Cos7 cells, and carried out co-immunoprecipitation (co-IP) experiments. We found little if any interaction with the wild type version of the GBF1 Sec7 domain, but found that the catalytically inactive mutant version (in which a key glutamic acid residue of the Sec7 domain is replaced with lysine, abolishing exchange activity [43]) gave positive results (Figure 1B). The expression of the wild type Sec7 domain likely interferes with activity of the corresponding endogenous protein, and is not well tolerated by cells. Using the EK mutant versions, all of the Arf1-specific Sec7 domains interacted with ATGL in co-IP experiments, but not the Sec7 domain of EFA6 (Figure 1C). Hence by co-IP, like in the yeast two-hybrid assay, ATGL did not interact with an Arf6-specific Sec7 domain, but among Arf1-specific Sec7 domains, interaction was promiscuous.

**Figure 2:**
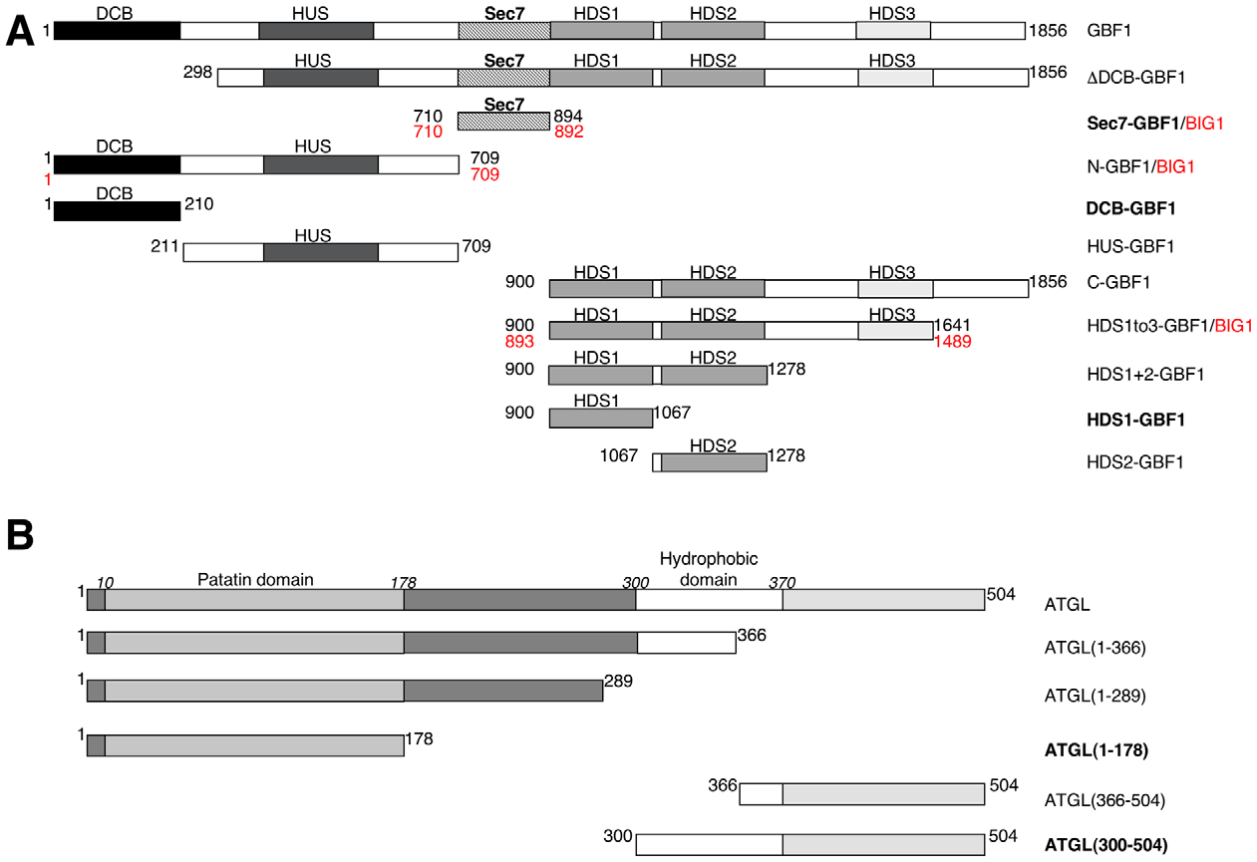
Fragments of *Homo sapiens* GBF1 and ATGL proteins used in this study. A- Schematic diagram of GBF1 showing domains, with regions used in the interaction experiments shown below. Amino acid numbers for the beginning and end of each fragment are indicated. Numbers in black are for GBF1, numbers in red refer to BIG1. B- Schematic diagram of ATGL, with regions used in interaction experiments shown below. The constructs indicated in bold are those that were demonstrated to interact directly when expressed in *E. coli* and were also shown to interact by co-immunoprecipitation and yeast two-hybrid methods (with the exception of the HDS1-ATGL patatin domain interaction which was not detected in yeast two-hybrid); see Table 2 for details. Note that slight variations in the borders of GBF1 constructs were used for the yeast two-hybrid experiments (see Table 1), but essentially the same domains were used for all interaction studies.

Our previous results have shown that GBF1 functions in delivery of ATGL to LDs, suggesting that GBF1 might be the physiological partner of ATGL [38]. The other large Arf1 GEFs, BIG1 and BIG2, are highly homologous (74% identical in amino acid sequence), and have many overlapping functions [7], [44]. To determine whether BIG1 and BIG2 might have functional connections to ATGL, we first looked for interactions between the regions of GBF1 and BIG1 flanking the Sec7 domain. We expressed either the N- or C-terminal regions of GBF1 and BIG1 with ATGL in Cos7 cells, and tested interaction by co-IP. The N-GBF1 and N-BIG1 constructs contain the entire region of GBF1 or BIG1, respectively, upstream of the Sec7 domain, and the C-terminal GBF1 and BIG1 constructs contain the region spanning domains HDS1, HDS2 and HDS3 downstream of the Sec7 domain (HDS1to3) (Figure 2). The expression levels of these constructs in Cos7 cells are shown in Figure S1A, B. Interaction of these domains was observed with GBF1, but not with the equivalent domains of BIG1 (Figure S2A). Next, we tested whether depletion of BIG1 and BIG2 played a role in delivery of ATGL to LDs. Knocking down GBF1 in HeLa cells inhibited localization of ATGL to LDs, as shown previously [38], but knocking down BIG1 and BIG2 either individually (data not shown) or both together (Figure S2B-D) had no effect. These results strongly support the hypothesis that the functional connection between GBF1 and ATGL is specific to GBF1 (at least among the large Arf1 GEFs) and that regions outside the Sec7 domain are important for specific interaction between GBF1 and ATGL.

### Mapping Interaction Domains of ATGL and GBF1

**Figure 3:**
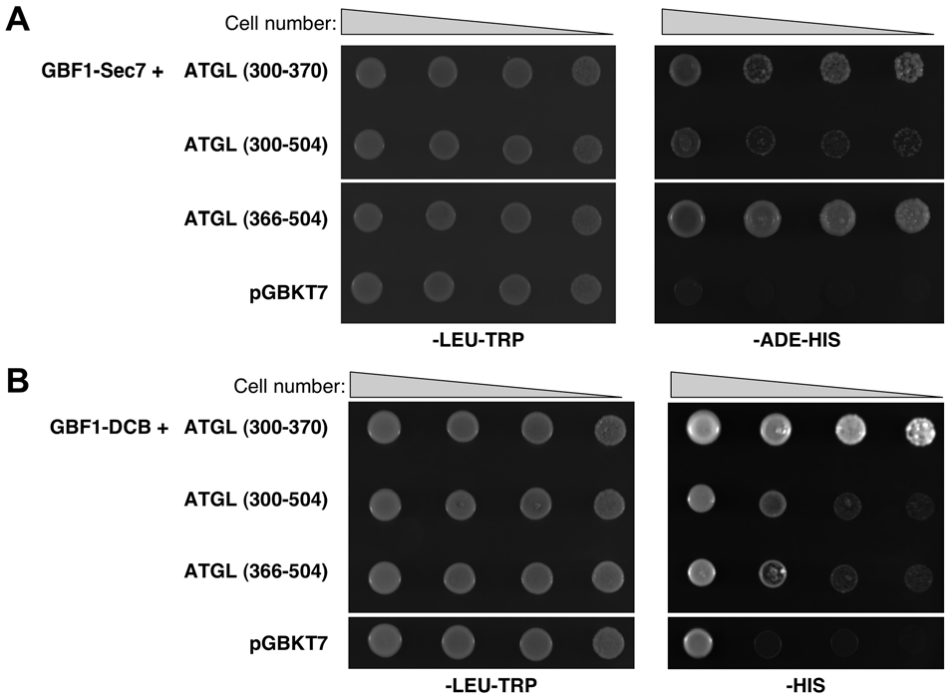
Yeast two-hybrid interactions between ATGL regions and GBF1 Sec7 and DCB domains. Yeast strain AH109 was transformed with the indicated GBF1 domains in pGADT7, along with the indicated regions of ATGL in pGBKT7 plasmids. 10-fold serial dilutions of each doubly transformed strain were spotted onto the indicated plates and incubated for 2 days at 30°C. A- GBF1 Sec7 domain. B- GBF1 DCB domain.

We carried out yeast two-hybrid and co-IP experiments to map the domains of interaction between GBF1 and ATGL. A diagram of the domains we have used for each protein is shown in Figure 2. For co-IP experiments, the levels of expression of each in Cos7 cells are shown in Figure S1. With the GBF1 Sec7 domain, in addition to the interaction between the C-terminal region of ATGL identified in the yeast two-hybrid screen, we found an interaction with the hydrophobic domain just upstream (amino acids 300–370) (Figure 3A). We also identified an interaction between the Dimerization/Cyclophilin Binding (DCB) domain of GBF1 and this C-terminal hydrophobic domain of ATGL, as well as a weaker interaction with the C-terminal region downstream (amino acids 366–504) (Figure 3B). A summary of the yeast two-hybrid interaction data is shown in Table 1.

**Table 1:** Yeast two-hybrid interactions between regions of GBF1 and ATGL.

	ATGL							
GBF1	Full-length (M1-L504)	Patatin (M1-L178)	M1-M366	M1-L252	M366-L504	L300-L504	L300-T370	pGBKT7
DCB (V2-E202)	0	0	0	0	+	+	++	0
HUS* (P203–P363)	0	0	0	0	0	0	0	0
HUS-D544A* (P203–P363)	0	0	0	0	0	0	0	0
N (M1-N698)	0	0	0	0	0	0	0	0
N-D544A (M1-N698)	0	0	0	0	0	0	0	0
Sec7 (F710-V894)	0	0	0	0	+++	+	++	0
C (N896-S1856)	0	0	0	0	0	0	0	0
pGADT7	0	0	0	0	0	0	0	0

The indicated GBF1 fragments (first column) are in vector pGADT7 (amino acids at the beginning and end of each fragment are given), and the indicated portions of ATGL (first row) are in pGBKT7. Level of growth on –HIS-LEU-TRP and –ADE–HIS-LEU-TRP selective plates is indicated by plus signs (+); “0” indicates no growth on selective plates. *Note that these constructs differ from the HUS-GBF1 domain shown in Figure 2 in that the region between the HUS and Sec7 domains is not present.

**Figure 4:**
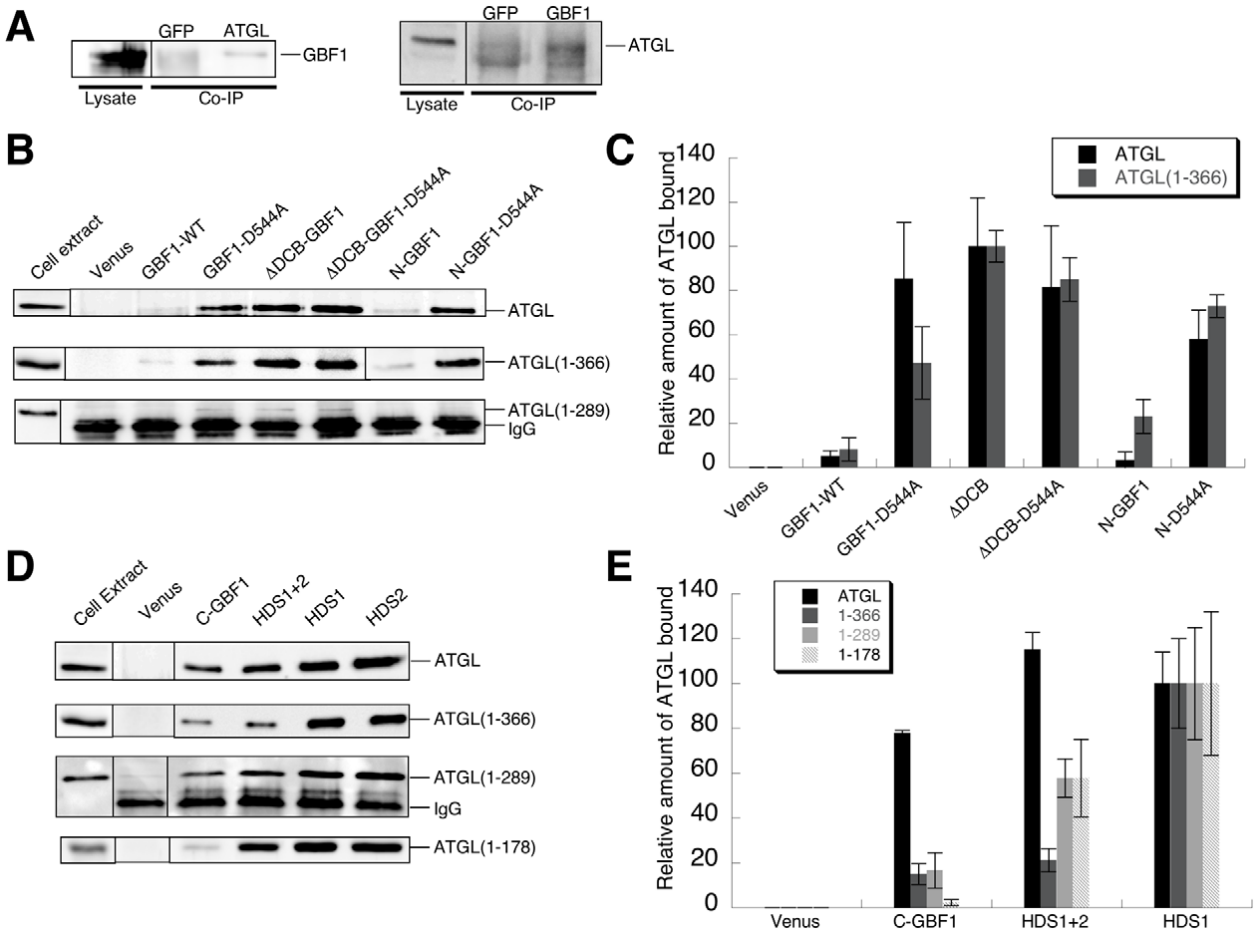
Interactions between GBF1 and ATGL assayed by co-immunoprecipitation. A- Immmunoprecipitation (IP) of endogenous proteins. In the left panel, ATGL antibody (GFP antibody for the negative control) was used for the IP, and the western blot was performed with GBF1 antibody. In the right panel, GBF1 antibody (GFP antibody for negative control) was used for the IP, and the western blot was performd with ATGL antibody. B- HA-tagged *H. sapiens* ATGL (either full-length or C-terminally truncated, as indicated) was coexpressed in Cos7 cells either with Venus alone, or with the indicated Venus-tagged GBF1 region. Immunoprecipitation was carried out with anti-GFP antibodies, and Cos7 cell lysates or eluted proteins after immunoprecipitation were analyzed by Western blotting using anti-HA antibody. For Western blots of the GFP-immunoprecipitated bait proteins, see Figure S3A. C- Quantification represents the level of ATGL in the indicated immunoprecipitate, after subtracting the value for pVenus and normalizing to the fragment with the highest level of interaction, that of the ΔDCB construct. This value was set to 100% for each ATGL construct; as shown in part B, these values were equivalent for full-length ATGL and ATGL(1–366). Mean and standard deviation of 2–4 independent experiments are shown. D- Co-immunoprecipitations from Cos7 cells expressing the indicated portion of HA-tagged *H. sapiens* ATGL and Venus-tagged GBF1 C-terminal regions were carried out as in part B. E- Quantifications were carried out as in part C, with values normalized to ATGL levels in HDS1 immunoprecipitations. Mean and standard deviation of 2–4 independent experiments are shown. Quantifications were carried out only for the three GBF1 fragments whose expression levels were approximately equivalent; HDS2 was expressed at a significantly higher level (see Figure S1A).

In co-IP experiments, we first tested the interaction between endogenous GBF1 and ATGL proteins (Figure 4A). The interaction was clear and reproducible, but appeared to be quite weak, so we next tested interaction between tagged full-length GBF1 and full-length ATGL expressed from plasmids. In this case as well, we saw a weak interaction (Figure 4B; quantifications shown in Figure 4C). For another interacting partner of GBF1, γ-COP, we also found a very weak interaction with the full-length GBF1 protein [5]. In this case, interaction was stronger with mutant and truncated versions of GBF1 that disrupt an intramolecular interaction between the DCB and Homology upstream of Sec7 (HUS) domains [5], [45]. Introduction of a mutation in the HUS domain of GBF1 that disrupts the DCB-HUS interaction (D544A) led to a significant increase in the level of interaction with ATGL (Figure 4B, C). Note that this mutation does not change the level of expression of GBF1 (Figure S1A) nor the amount of protein immunoprecipitated (Figure S3A). When the entire DCB domain of GBF1 was deleted, interaction with ATGL was stronger as well (Figure 4B). In both cases, the level of interaction was about 10-fold higher than for wild type GBF1 (Figure 4C), with 2–4% of ATGL in cell lysates recovered in association with these forms of GBF1. Similar results were obtained for the N-terminal region of GBF1 alone: little interaction with ATGL for the wild type form, and a 5–10-fold higher level for the D544A mutant version (Figure 4B, C). We also observed interaction of ATGL with the GBF1 DCB and HUS domains alone (Figure S3B). These results indicate that the N-terminus of GBF1 interacts with ATGL, and support the conclusion that the intramolecular interaction between the DCB and HUS domains inhibits this interaction.

The interaction of the N-terminally truncated form of GBF1 was stronger than with the mutant N-terminus alone, suggesting further interaction sites in the C-terminus of the protein. Indeed, we were able to detect interaction of the GBF1 HDS1 and HDS2 domains with ATGL (Figure 4D, E). These interactions were quite strong in co-IPs, with approximately 10% of ATGL in the co-IPs recovered in association with the HDS1 and/or HDS2 domains of GBF1 (Figure 4D, E).

We next tested deleted forms of ATGL for interaction with GBF1 in co-IP experiments. Deletion of the C-terminal region of ATGL (366–504) did not affect the interactions with the N- and C-terminal regions of GBF1 (Figure 4B–E, Figure S3B), consistent with this region interacting primarily with the Sec7 domain (Figure 1, 3B). Mutations in ATGL are linked to the human disease NLSD with myopathy [46]. Three disease alleles lead to truncation of the ATGL protein, including one truncation at amino acid 289. Previous results have shown that these mutants have higher lipase activity than the wild type protein *in vitro* [22]. ATGL(1–289) showed little if any interaction with the N-terminal domains of GBF1 in co-IP experiments (Figure 4B). However, ATGL(1–289) interacted well with the C-terminal HDS1 and HDS2 domains of GBF1 (Figure 4D, E). Further truncation of ATGL indicated that the minimal lipase domain (the patatin domain) alone interacted with the GBF1 HDS1 and HDS2 domains. Hence the co-IP experiments confirm the yeast two-hybrid results indicating that interactions with the N-terminus of GBF1 occur primarily with the C-terminal regions of ATGL (amino acids 300–504), and further show that the GBF1 HDS1 and HDS2 domains interact with the N-terminal patatin domain of ATGL (amino acids 1–178).

**Figure 5:**
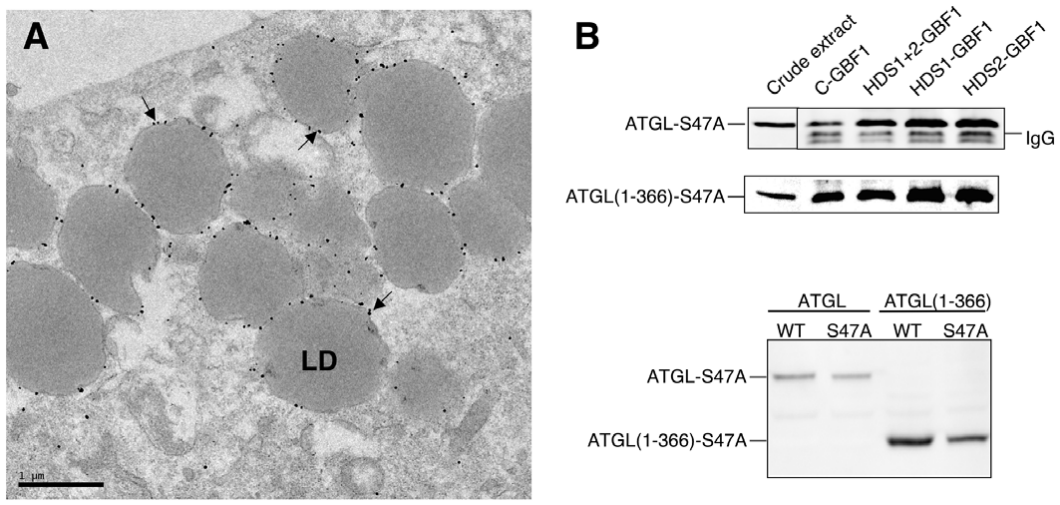
Catalytic activity of ATGL is not required for LD localization or interaction with GBF1. **A-** Immuno-EM of HeLa cells expressing GFP-tagged *H. sapiens* ATGL-S47A were treated with 200 µM oleic acid for 15 hours and labeled with gold-conjugated antibodies against GFP. The majority of gold particles (examples indicated by arrows) are found on the surface of LDs. LD, lipid droplet. **B-** Co-immunoprecipitations from Cos7 cells expressing HA-tagged *H. sapiens* ATGL-S47A or ATGL(1–366)-S47A and Venus-tagged GBF1 fragments were carried out as in Figures 1 and 4; Western blot of immunoprecipitates probed with anti-HA antibodies (upper panel); Cos7 cell extracts blotted with anti-GFP antibodies (lower panel).

To determine whether catalytic activity of ATGL was important for its interaction with the HDS1 and HDS2 domains of GBF1, we expressed the S47A mutant, known to abolish lipase activity [20]. Immuno-EM analysis indicated that this catalytically inactive mutant was expressed and localized properly to the surface of LDs (Figure 5A). ATGL-S47A interacted with the GBF1 HDS1 and HDS2 domains to an extent equivalent to the wild type ATGL protein (Figure 5B), indicating that catalytic activity is not required for interaction with GBF1. Interaction of the N-terminal domains of GBF1 were also not affected by the S47A mutation in ATGL (Figure S3C).

**Figure 6:**
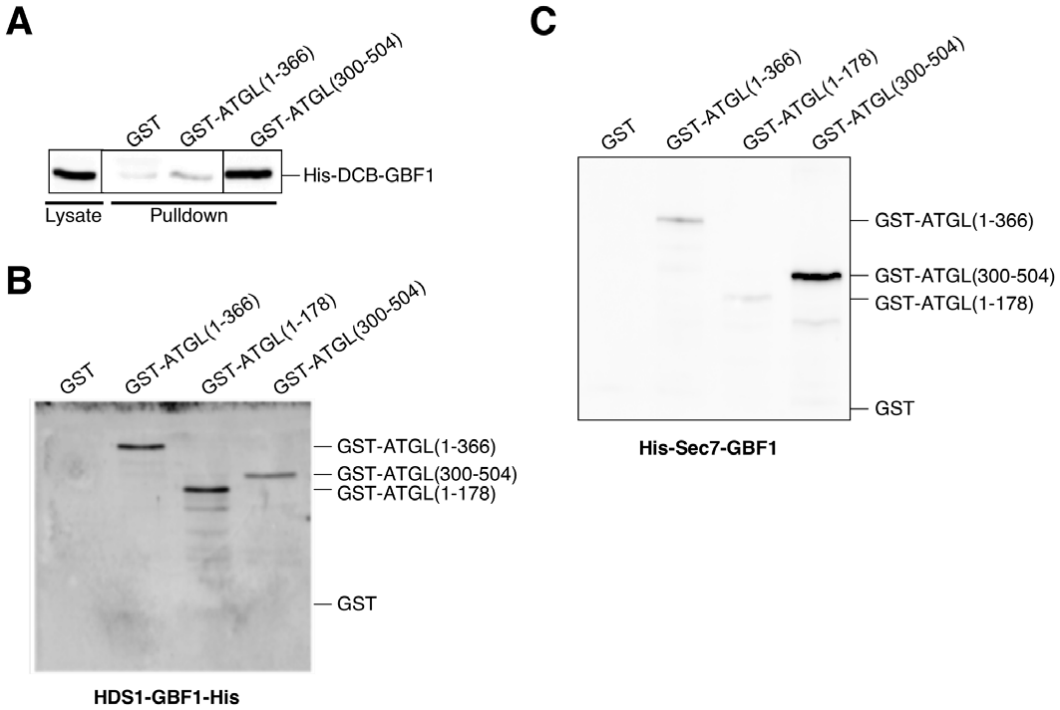
Direct interactions between domains of GBF1 and ATGL. **A-** Purified GST, GST-tagged ATGL(1–366) or GST-tagged ATGL(300–504) was bound to glutathione Sepharose beads, then incubated with *E. coli* lysates expressing the His-tagged DCB domain of GBF1. Eluted proteins were analyzed by Western blotting using His antibody. **B- and C-** The indicated His-tagged GBF1 domain expressed in *E. coli* was bound to Ni Sepharose 6 Fast Flow, then purified GST or the indicated GST-tagged domain of ATGL were incubated with the protein-bound beads. Eluted proteins were analyzed by Western blotting using GST antibody. **B-** The HDS1-His domain of GBF1 bound to Ni Sepharose beads. **C-** The His-tagged Sec7 domain of GBF1 bound to Ni Sepharose beads.

**Figure 7:**
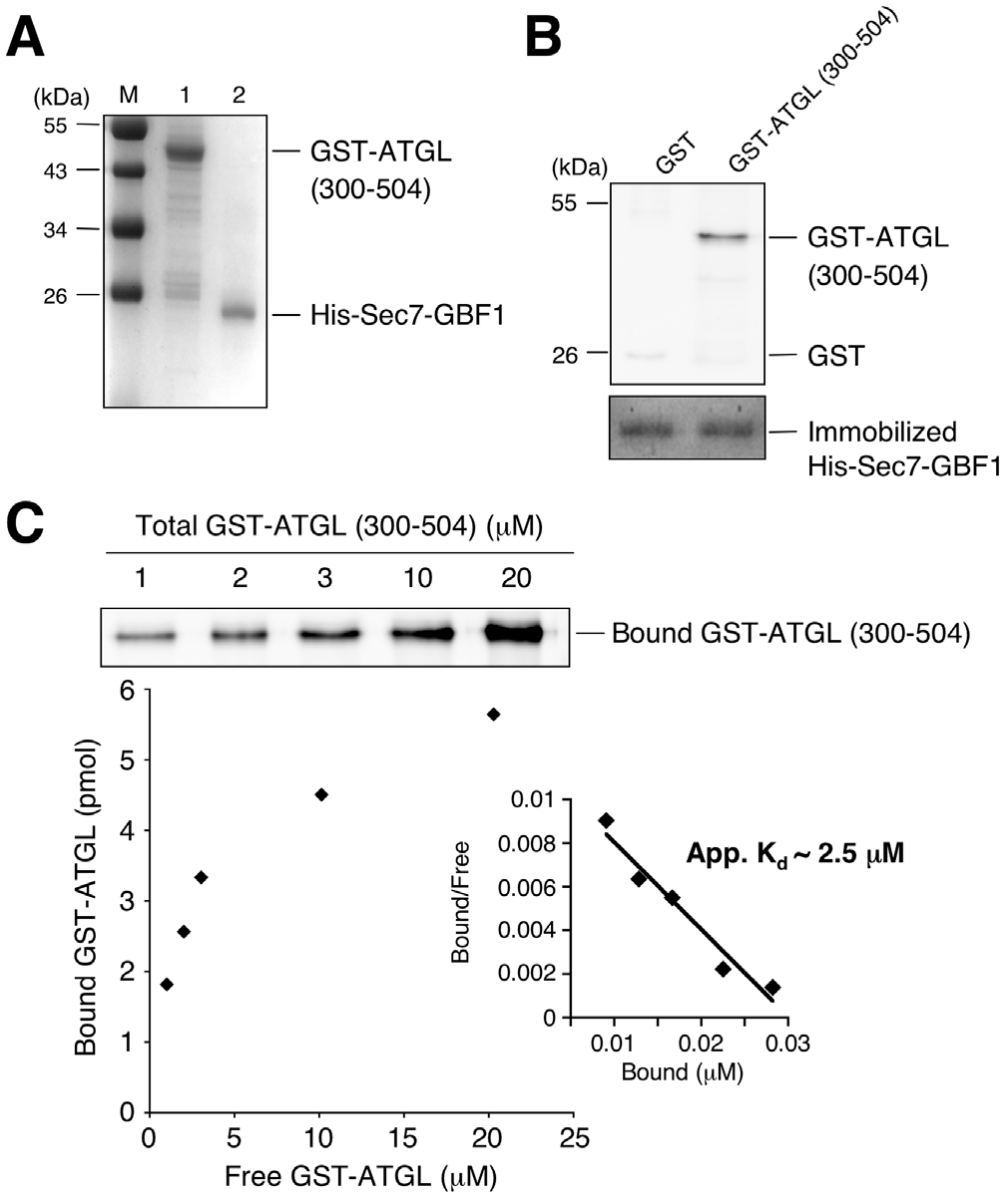
The GBF1 Sec7 domain directly interacts with the ATGL (300–504) domain in vitro. A- Coomassie stained gel showing purified recombinant GST-tagged ATGL (300–504) (1 µg, lane 1) and His-Sec7-GBF1(710–894) (1 µg, lane 2). M, molecular weight markers. B- 0.1 nmole of His-Sec7-GBF1(710–894) were immobilized on a Ni Sepharose 6 Fast Flow resin, then incubated with either GST or GST-tagged ATGL(300–504) (0.5 µM each) for 2.5 hours at 4°C. After washes, proteins were eluted, separated by SDS-PAGE followed by immunoblotting with anti-GST antibody (upper panel) or by staining with Coomassie Brilliant Blue (lower panel). GST-tagged ATGL(300-504) specifically bound to the His-Sec7-GBF1 fragment. C- Quantitative analysis of the interaction between His-tagged GBF1 Sec7 domain and GST-tagged ATGL (300–504) was performed. Various concentrations (1–20 µM) of GST-tagged ATGL (300–504) were added to immobilized His-Sec7-GBF1(710–894) domain (0.1 nmole). Each sample was washed and subjected to SDS-PAGE followed by immunoblotting with anti-GST antibody (upper panel). The amount of bound versus free GST-tagged ATGL (300–504) domain is plotted (lower panel). Scatchard analysis indicated an apparent K_d_ of 2.5±0.3 µM (inset).

To determine whether the interactions between GBF1 and ATGL domains were direct, we expressed individual domains of both proteins in E. coli and tested binding. We found evidence for three direct interactions. Approximately 1% of the DCB domain of GBF1 in an E. coli extract interacted with the purified GST-ATGL (300–504) C-terminal fragment (Figure 6A). This interaction was specific, as no interaction was detected with the N-terminal region of ATGL (1–366). These results are in good agreement with the yeast two-hybrid results (Figure 3B). We also observed a direct interaction between the N-terminus of ATGL and the HDS1 domain of GBF1 (Figure 6B). Finally, we observed a direct interaction between the C-terminus of ATGL and the Sec7 domain of GBF1 (Figure 6C). We measured an apparent Kd of 2.5±0.3 µM for this interaction (Figure 7). A summary of the major interactions between the different domains of GBF1 and ATGL are given in Table 2.

**Table 2:** Summary of major interactions between GBF1 and ATGL.

Interaction	Methods used	Comments
GBF1 Full-lengthATGL Full-length	Co-IP:- Endogenous proteins- Tagged proteins expressed from plasmids	Interaction between full-length proteins is stronger when GBF1 HUS-DCB interaction is disrupted (by D544A mutation in HUS domain or deletion of DCB domain).
GBF1 DCB domainATGL C-terminus (aa300–504)	- Yeast 2-hybrid- Co-IP tagged fragments- Purified proteins	The GBF1 DCB domain interacts best with the hydrophobic domain (aa300–370) of the ATGL C-terminus.
GBF1 Sec7 domainATGL C-terminus (aa300–504)	- Yeast 2-hybrid- Co-IP tagged fragments- Purified proteins	Direct interaction with an apparent Kd of approximately 2.5 µM.
GBF1 HDS1 domainATGL patatin domain (N-term)	- Co-IP tagged fragments- Purified proteins	In co-IP experiments, GBF1 HDS2 also interacts with ATGL patatin domain.

### Localization of GBF1 HDS1 and HDS2 domains and ATGL truncations to Lipid Droplets

**Figure 8:**
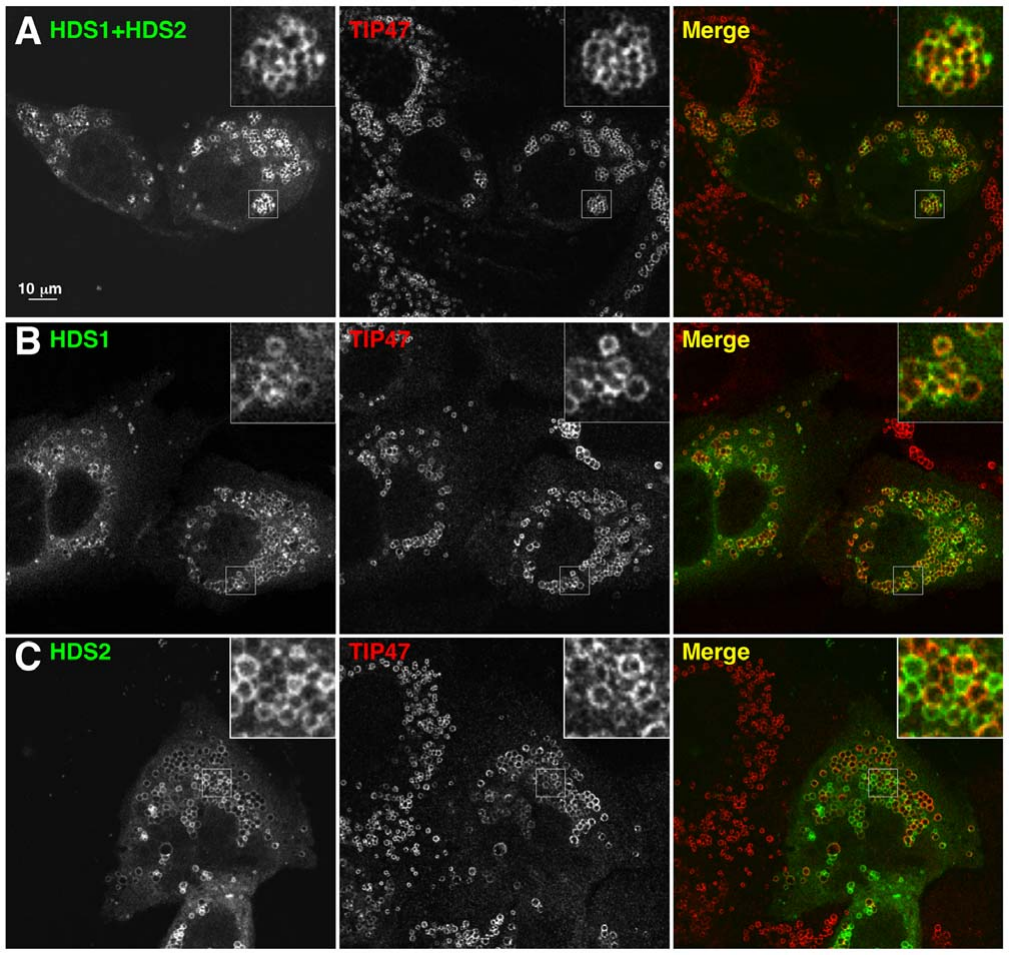
The GBF1 HDS1 and HDS2 domains localize to lipid droplets in cells. HeLa cells transfected with Venus-tagged HDS1 and/or HDS2 domains of GBF1 as indicated were treated with 400 µM oleic acid for 3 hours, then immunostained with antibodies against TIP47 (middle panels). Venus fluorescence is shown on the left; the merge image on the right. Bar: 10 µm. **A-** Cells expressing the region of GBF1 from the beginning of HDS1 to the end of HDS2, N-terminally tagged with Venus. **B-** Cells expressing the Venus-tagged HDS1 domain. **C-** Cells expressing Venus-HDS2. Bar, 10 µm.

As a first approach to understanding the physiological relevance of the multiple interactions between domains of GBF1 and ATGL that we demonstrated above, we sought to determine whether any domains of GBF1 had a specific localization to LDs. Full-length GBF1 localizes to both the Golgi complex and to LDs [38]. Preliminary localization studies in HeLa and Cos7 cells with different domains of GBF1 showed that the N-terminus localized predominantly to the cytosol, and that the entire C-terminal region downstream of the Sec7 domain partially localized to LDs, although there was also a large cytosolic pool (data not shown). To determine whether the C-terminal HDS1 and HDS2 domains of GBF1 are involved in localization to LDs, we expressed these domains as fusions to GFP in HeLa cells. Each domain alone, and a construct carrying both domains, localized to LDs (Figure 8). Interestingly, and in contrast to wild type GBF1, these HDS1 and HDS2 constructs did not localize to the Golgi (Figure 8).

**Figure 9:**
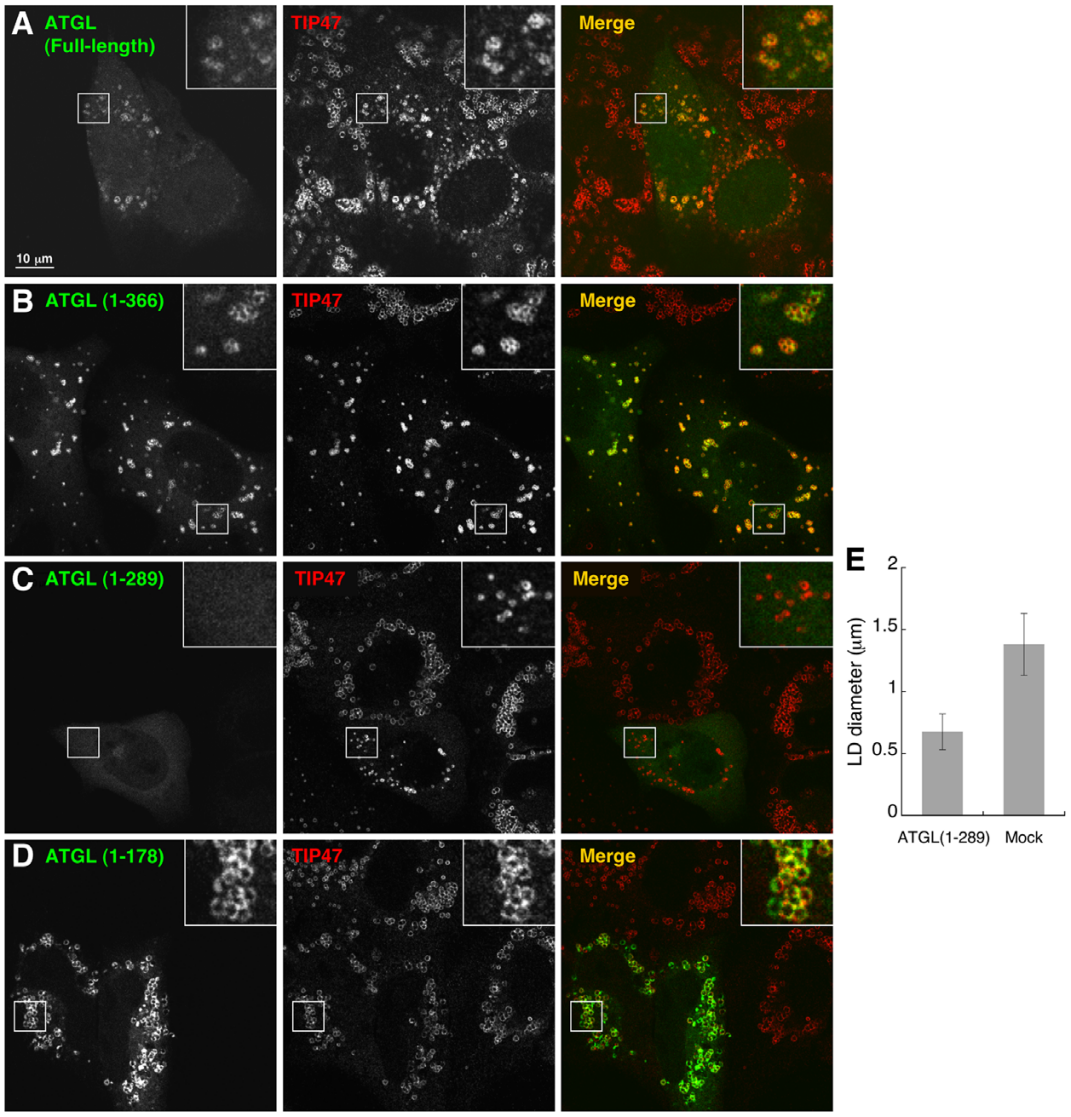
The C-terminal region of ATGL regulates its localization to LDs in cells. HeLa cells transfected with the indicated ATGL constructs tagged at their C-terminus with GFP were treated with 400 µM OA for 3 hours, then immunostained with antibodies against TIP47 (middle panel). GFP fluorescence is shown on the left; the merge image on the right. Bar: 10 µm. **A-** Cells expressing full-length ATGL(1–504)-GFP. **B**- Cells expressing ATGL(1–366)-GFP. **C-** Cells expressing ATGL(1–289)-GFP. **D-** Cells expressing ATGL(1–178)-GFP. **E-** LD diameters were measured in cells expressing ATGL(1–289)-GFP and in control cells. Mean and standard deviation of three independent experiments are shown. Bar, 10 µm.

We expressed different truncations of ATGL, with GFP fused to their C-termini, in HeLa cells to test their localization. Full-length ATGL with GFP appended to its C-terminus is functional, and results in a decrease in size of LDs when overexpressed [20] (Figure 9A). We found that truncation of ATGL after amino acid 366 did not affect its localization to LDs, and like the wild type protein, lipid droplets were smaller upon overexpression of this C-terminally truncated form (Figure 9B). The truncated version of ATGL corresponding to that expressed in NLSD disease patients, ATGL(1–289), had a very interesting behavior. Consistent with its high level activity in vitro [22], this truncation expressed in HeLa cells resulted in a significantly reduced size of LDs (Figure 9C, E). However, ATGL(1–289) did not accumulate on these smaller LDs (Figure 9C), in contrast to the longer version, ATGL(1–366) (Figure 9B). Further truncation of ATGL, containing the patatin domain, ATGL(1–178), did accumulate on LDs but did not lead to a decrease in their diameter (Figure 9D). Hence the N-terminal region of ATGL containing the patatin domain is sufficient for localization to LDs, but the region downstream, between amino acids 178 and 366, appears to be involved in regulating interaction of ATGL with LDs.

## Discussion

We report here an interaction between the triglyceride lipase ATGL and the Arf1 GEF GBF1. We found an interaction between the endogenous proteins in mammalian cells by co-immunoprecipitation, and sought to map their interaction domains. Several regions of each partner are involved in this interaction, which appears to be quite complex. Although we originally identified ATGL as a binding partner of the Sec7 domain of the BIG2 Arf1 GEF, we do not find evidence for a function of BIG1 or BIG2 in delivery of ATGL to LDs. A similar situation has been reported recently for the yeast homologues of GBF1 and BIG1/2, Gea2p and Sec7p, respectively. An interaction between the Pik1p phosphoinositide-4 kinase and the Sec7 domain of the Arf1 GEF Gea2p was identified in a yeast two-hybrid screen [47]. Interactions between Pik1p and Arf1-specific Sec7 domains (those of Gea2p and Sec7p) were promiscuous, and the originally identified Sec7 domain protein (Gea2p) was apparently not the physiologically relevant partner, but rather Sec7p was the GEF functionally linked to Pik1p [47]. Previously, we found an interaction between the Gea2p Arf1 GEF and Drs2p, an amino-phospholipid translocase (flippase) at the yeast TGN [48], [49]. Hence an emerging theme is functional connections between the large Arf1 GEFs and lipid modifying enzymes, highlighting the importance of these Arf1 activators in directly regulating lipid metabolism in cells.

The interaction of GBF1 with ATGL appears to have similarities to GBF1 interaction with the COPI subunit γ-COP [5]. There is only a weak interaction with wild type GBF1, but if the intramolecular interaction between the DCB and HUS domains is compromised through mutation or deletion, a robust interaction is revealed. Our data are consistent with GBF1 being in an initially closed conformation that interacts poorly with binding partners, and that then becomes “opened up” to expose the DCB and HUS domains for optimal interaction. Our domain mapping experiments, using yeast two-hybrid, co-IP and expression of domains in *E. coli*, indicate that there are multiple interactions of ATGL with at least four domains of GBF1. The N-terminal DCB and Sec7 domains of GBF1 interact with the C-terminal region of ATGL, the GBF1 HDS1 and HDS2 domains interact with the N-terminal patatin domain of ATGL (Table 2). The Sec7 domain of GBF1 interacts well with region 366–504 of the C-terminus of ATGL, and the DCB domain interacts most strongly with region 300–370, which corresponds to a hydrophobic domain that has been postulated to interact directly with lipids and is required for localization of ATGL to LDs in cells [22], [50].

We found that the patatin domain of ATGL (amino acids 1–178) is targeted to LDs, but does not affect their size, unlike full-length ATGL or longer C-terminal truncations. This result suggests that ATGL (1–178) may not be active on LDs in HeLa cells. The C-terminal truncation of ATGL removing the region downstream of amino acid 366 has a behavior similar to that of the wild type protein, both localizing to LDs and leading to a decrease in their size. Our data support the conclusion that in addition to the patatin domain, region 178–289 of ATGL is required for ATGL activity on LDs, whereas region 289–366 (which interacts with the N-terminal regions of GBF1) is required for stable localization of this catalytically active portion of ATGL to LDs. These results are in excellent agreement with the work of Zimmermann and colleagues, who demonstrated that a mutation downstream of the patatin domain, P195L, completely abolished ATGL activity in vitro but retained the capacity to bind to LDs. Both the Zimmermann and Liu groups have shown that C-terminal truncations of ATGL fail to localize with LDs, and that the hydrophobic domain is required for LD localization [22], [50]. In addition, the C-terminus of ATGL (notably the region from amino acids 290–504) negatively regulates the lipase activity of ATGL *in vitro* [22]. The ATGL co-activator CGI-58 stimulates the activity of both the full-length and the C-terminally truncated forms of ATGL, but can stimulate the full-length autoinhibited form of ATGL to only a limited extent [22]. Hence other factors, such as GBF1, might be required to relieve the autoinhibition mediated by the C-terminal region of ATGL.

The HDS1 and HDS2 domains of GBF1 localize on their own to LDs when expressed in HeLa cells, and strikingly, do not localize at all to the Golgi. ATGL and other LD-associated proteins have a similar localization: there is no visible accumulation of LD proteins at the Golgi [38], [51]. This result suggests that there is a sorting event that takes place in a pre-Golgi compartment whereby GBF1 is directed either towards LDs or to the Golgi. The regions flanking the HDS domains in GBF1 may play a role in directing GBF1 to the Golgi, with trafficking to LDs representing a type of default pathway. We found previously that ATGL is very tightly associated with membranes, and when its trafficking to LDs is blocked, it accumulates in structures at the ER containing COPII, which correspond to ER export sites (ERES) [38]. ERES mature to form ERGIC elements upon recruitment of GBF1 and its activation of Arf1 [8]. ERGIC elements, containing the marker ERGIC-53, detach from the ER and form a pre-Golgi compartment that has been referred to as the biosynthetic recycling compartment (BRC) [52]. Saraste and Goud have proposed that this compartment, parallel to the endocytic recycling compartment (ERC), acts as a sorting station for multiple pathways, including anterograde transport of secretory proteins to the Golgi and retrograde recycling back to the ER [52]. We showed previously that the GBF1-dependent pathway that delivers ATGL to LDs includes ERGIC-53 but not the Golgi protein GM130 [38], suggesting that it bifurcates from the secretory pathway at the level of the ERGIC/BRC. Since GBF1 functions in both trafficking to the Golgi and to LDs [3], [38], [39], we propose that the GBF1-ATGL interaction might be involved in a sorting step at the pre-Golgi BRC compartment that directs ATGL and the GBF1-Arf1-COPI machinery to LDs.

## Materials and Methods

### Strains, plasmids and antibodies

Plasmids used in this study are listed in Table S1. Point mutations were introduced using the QuickChange II XL site-directed mutagenesis kit (Stratagene). All constructs were confirmed by DNA sequencing. pGBKT7-ATGL(300–370), pGBKT7-ATGL(300–504), and pGBKT7-ATGL(366–504) were constructed by insertion of the ORF fragment amplified by PCR into EcoRI and SalI sites of pGBKT7 (Clontech). pGEX-4T1-ATGL(1–366), pGEX-4T1-ATGL(300–504), and pGEX-4T1-ATGL(1–178) were constructed by insertion of the ORF fragment amplified by PCR into EcoRI and SalI sites of pGEX-4T1 (GE Healthcare). pcDNA-ATGL, pcDNA-ATGL(1–366), pcDNA-ATGL(1–289), and pcDNA-ATGL(1–178) were constructed by insertion of the ORF/ORF fragment amplified by PCR into BamHI and XhoI sites of pcDNA3-HA (Invitrogen). pEGFP-N1-ATGL, pEGFP-N1-ATGL-S47A, pEGFP-N1-ATGL(1–178), pEGFP-N1-ATGL(1–289), pEGFP-N1-ATGL(1–366), pEGFP-N1-ATGL(1–366)-S47A were constructed by insertion of the ORF/ORF fragment amplified by PCR into BamHI and KpnI sites of pEGFP-N1 (Clontech). pcDNA-ATGL-S47A and pcDNA-ATGL(1–366)-S47A were constructed by subcloning from the corresponding pEGFP-N1 plasmid into SalI and BamHI sites of pcDNA3-HA.

pVenus-C1 was constructed by removing the Venus coding region from pVenus-N1 with AgeI and BspEI, and inserting this fragment into pEYFP-C1 digested with the same enzymes. The isoform of GBF1 used in this study has been described previously [53]. pVenus-ΔDCB-GBF1 was constructed by digesting pVenus-GBF1 with EcoRI, removing the fragment, and recircularizing the gapped plasmid. Other pVenus plasmids expressing fragments of GBF1, BIG1, BIG2, ARNO, or EFA6 were constructed by insertion of the ORF fragments amplified by PCR into the XhoI and KpnI sites of pVenus-C1. pBAD plasmids were constructed by subcloning from pVenus plasmids into XhoI and KpnI sites of pBAD/His C (Invitrogen). pET22b-HDS1-GBF1 was constructed by insertion of the fragments amplified by PCR into the EcoRI and SalI sites of pET22b (Novagen). The GST fusion proteins and GBF1(908–1065)-His were produced using *E. coli* strain BL21(DE3) (Novagen). Other His-tagged proteins were produced using *E. coli* strain TOP10 (Novagen). pGBKT7-Sec7-BIG2 was constructed by insertion into sites EcoRI and SalII, and pGBKT7-Sec7-GBF1, pGBKT7-Sec7-EFA6 and pGBKT7-Sec7-ARNO were constructed by insertion into EcoRI and BamHI sites of pGBKT7 (Clontech). pGADT7-Sec7-GBF1 and pGADT7-DCB-GBF1 were constructed by insertion into EcoRI/BamHI sites and EcoRI/NcoI sites, respectively, of pGADT7 (Clontech). For the plasmids listed in Table 1, the indicated regions of ATGL were cloned into the EcoRI and SalI sites of pGBKT7; all GBF1 fragments were cloned into pGADT7 using the following sites: DCB-GBF1 (V2E202) EcoRI/NcoI, LinkerHUS-GBF1 (P203P363) SfiI/XhoI, N-GBF1 (M1N698) NcoI/XhoI and C-GBF1 (N896S1856) EcoRI/XhoI.

The following mouse monoclonal antibodies were used: clone HA-7 ascites fluid (Sigma) against the HA tag, clone GST-2 ascites fluid (Sigma) against GST, anti-GFP (Roche Diagnostics), QIAexpress Anti-His antibodies (Qiagen) against the His tag and guinea pig anti-TIP47 from Fitzgerald Industries International (Concord, MA, USA). ATGL [20] and GBF1 [5] antibodies have been described.

### Yeast two-hybrid assays

Yeast two-hybrid experiments were carried out as described previously [5]. The yeast two-hybrid screen was carried out using a human brain cDNA library (Clontech) with the pGBKT7-Sec7-BIG2 as bait, in strain AH109. 3×10^6^ clones were screened, 1843 clones picked after plating the library on selective plate (medium stringency), and after multiple tests of growth on -Ade-His-Trp-Leu selective plates, 132 clones were extracted from yeast cells and inserts sequenced.

### Expression of recombinant proteins in Escherichia coli

GST-tagged proteins were expressed in BL21(DE3) *E. coli* (Novagen) in 2xTY medium (GST-ATGL(300–504), GST-ATGL(1–178)) or in LB medium (GST, GST-ATGL(1–366)) induced with IPTG at 37°C for 4 hr (GST) or 28°C for 4 hr (GST-ATGL(1–366) and GST-ATGL(1–178)) or 2.5 hours (GST-ATGL(300–504). Cells were sonicated in TEX buffer (20 mM Tris-HCl pH 7.5, 30 mM KCl, 2 mM EDTA, 10% glycerol, 0.1 mM β-mercaptoethanol) in the presence of Complete Mini Protease Inhibitor cocktail (Roche Diagnostics) then centrifuged at 4°C. Proteins were purified on glutathione Sepharose 4B resin (GE Healthcare). Eluted proteins were dialyzed against 50 mM Tris-HCl pH 7.5, 30 mM NaCl, 1 mM DTT buffer, concentrated with Vivaspin system and loaded on a HiPrep 26/60 Sephacryl S-200 HR column. Eluted proteins were concentrated with Vivaspin system and dialyzed against the same buffer containing 50% glycerol. Proteins were kept at −20°C.

His-tagged proteins with the exception of GBF1(908–1065)-His were expressed in TOP10 cells (Novagen) in 2xTY medium at 37°C, and induced with arabinose. GBF1(908–1065)-His expression was performed in BL21(DE3) cells cultured in 2xTY medium at 37°C, and induced with IPTG. Cells were lysed in TEX buffer in the presence of lysozyme, DNaseA and Complete Mini Protease Inhibitor cocktail (Roche Diagnostics) or protease inhibitor mix (GE Healthcare) then centrifuged at 4°C.

### Pulldown assays

For the experiments involving HDS1-GBF1(908–1065)-His and His-Sec7-GBF1(710–894), *E. coli* extracts were incubated with Ni Sepharose 6 Fast Flow (GE Healthcare) at 4°C for 1 hr, with rotation. Beads were washed with washing buffer W250 (20 mM Tris-HCl pH 7.5, 250 mM NaCl), then were incubated with purified GST fusion proteins with rotation at 4°C for 1 hr. Beads were washed three times with W250. Bound proteins were eluted by incubation with 50 µL of SDS-PAGE sample buffer and heating at 95°C for 5 min, then were resolved by SDS-PAGE and revealed by immunoblotting using antibodies to GST-protein followed by chemiluminescent detection (GE Healthcare).

For the experiments involving His-DCB-GBF1(1–210), purified GST-tagged proteins were incubated with Glutathione Sepharose 4B beads (GE Healthcare). Beads were washed two times with washing buffer W250+ (20 mM Tris-HCl pH 7.5, 250 mM NaCl, 1 mM EDTA). Then, cellular extracts expressing His-tagged proteins were incubated at 4°C for 1 hr. Beads were washed four times with W250+ then one time with PBS. Bound proteins were eluted as described above, then were resolved by SDS-PAGE and revealed by immunoblotting using His-tag antibodies followed by chemiluminescent detection (GE Healthcare).

### Co-immunoprecipitation assays

Typically, Cos7 cells (ATCC) cultured in 3.5 cm culture dishes were cotransfected using Lipofectamine (Invitrogen, Carlsbad, CA, USA) with a pcDNA plasmid expressing a sub-region of HA-tagged ATGL and either a pVenus or a pEYFP plasmid expressing *H. sapiens* GBF1, BIG1, BIG2, ARNO or EFA6 (either full-length or a sub-region) fused to Venus or YFP. After 20 hours of expression, cells were washed two times with 5 mL of cold PBS, then disrupted in 0.1 mL of cold Lysis buffer (50 mM Tris-HCl pH 7.5, 100 mM NaCl, 1 mM EDTA, 0.5% NP40). After centrifugation at 4°C, cellular extracts were incubated with 0.8 µg of anti-GFP for 1.5 hours at 4°C with rotation. The resin was washed three times with 1 mL of W100 buffer (20 mM Tris-HCl pH 7.5, 100 mM NaCl, 1 mM EDTA) followed by one wash with 1 mL of PBS. For co-immmunoprecipitation of endogenous proteins, a 10 cm diameter plate of Cos7 cells was lysed in 150 µL Lysis buffer with antiproteases, the IP carried out as above but using GBF1 (BD Biosciences) and ATGL (Cell Signaling) antibodies, then the resin washed three times with W100 buffer. Proteins were then eluted by incubation with 50 µL of SDS-PAGE sample buffer for 5 minutes at 95°C. Eluted proteins were separated by SDS-PAGE and analyzed by Western immunoblotting using anti-HA or anti-GFP, and visualized using ECL Advance (or ECL) Western Blotting Detection Kit (GE Healthcare). Membranes were imaged using a Luminescent Image Analyzer LAS-3000 (FujiFilm), and the levels of GBF1 and ATGL full-length proteins (both endogenous and ectopically expressed) and fragments were quantified using ImageJ software.

### Immunofluorescence and Microscopy

Oleic acid (OA) complexed to bovine serum albumin (BSA) was prepared as described [38]. HeLa cells were grown in Dulbecco's Modified Eagle's medium (DMEM) supplemented with 4.5 g/l glucose and sodium pyruvate, 20 mM glutamine, 100 units/ml penicillin G and 100 µg/ml streptomycin. HeLa cells (ATCC) were transfected with plasmids on 24-well plates using Lipofectamine (Invitrogen) according to the manufacturers' instructions. At 8–12 hours post transfection, cells were treated with 400 µM OA and incubated for 3 hours, then prepared for imaging. For overnight OA treatment, at 8 hours post transfection, cells were treated with 200 µM OA and incubated for an additional 15 hours before preparation for imaging. For immunofluorescence microscopy, HeLa cells were grown on glass coverslips and fixed with 4% formaldehyde. The cells were permeabilized with 0.1% saponin for 10 min, washed with PBS, then treated with 0.2% fatty-acid-free BSA and 0.2% gelatin in PBS for 15 min, followed by a 30 min wash in PBS. Cells were probed with primary and secondary antibodies for 1 hour followed by 30 min washes in PBS. To stain the LD cores, 10 µg/ml of BODIPY 493/503 (Invitrogen) was incorporated with the secondary antibodies. Coverslips were mounted on glass slides using Fluoromount G (Southern Biotechnology Associates). Images were acquired using an inverted confocal laser scanning microscope (LSM 510; Carl Zeiss). LD size quantifications were performed using the 510 Image Analyzer software. siRNAs and treatments were as described previously [5], [38].

HeLa cells were prepared for immuno-EM as described previously [54]. Briefly, cells were fixed in 4% paraformaldehyde, then blocked with 1% bovine serum albumin in PBS. Cells were permeabilized and incubated with primary GFP antibody (rabbit anti-GFP; Invitrogen) and subsequently incubated with nanogold-conjugated secondary antibodies (Nanoprobes). Cells were fixed with glutaraldehyde, treated with gold enhancement mixture for 6 min and postfixed in reduced osmium prior to embedding in Epon. 70–100 nm sections were cut and stained with lead citrate prior to imaging.

## Supporting Information

Figure S1 **Expression level of Venus-GBF1 and HA-ATGL constructs in Cos7 cells.** **A**- Lysates from Cos7 cells transfected with the indicated Venus-tagged GBF1 regions were analyzed by Western blotting using anti-GFP antibody. These results are representative of experiments carried out at least 3 times; co-expression of ATGL constructs did not affect GBF1 levels. The DCB-linker constuct contains the DCB domain plus the region between DCB and HUS domains (see Figure 2). **B**- Western blot using GFP antibodies of lysates from Cos7 cells transfected with the indicated Venus-tagged GBF1 or BIG1 construct. **C**- Western blot using HA antibodies of lysates from Cos7 cells transfected with the indicated HA-tagged ATGL construct.
(TIF)

Figure S2 **Specificity of interaction between ATGL and GBF1.** A- HA-tagged *H. sapiens* ATGL was coexpressed in Cos7 cells with the indicated Venus-tagged GBF1 or BIG1 region. Immunoprecipitation was carried out with anti-GFP antibodies, and eluted proteins after immunoprecipitation were analyzed by Western blotting using anti-HA antibody. B- to H- Depletion of BIG1 and BIG2 does not affect ATGL association with LDs. HeLa cells were transfected with siRNAs targeting BIG1 and BIG2 together (B-, E-, F-), GBF1 (C-, G-) or lamin (negative control) (D-, H-), then immunostained with antibodies against TIP47 (B-, C-, D-, upper panels) or ATGL (B-, C-, D-, lower panels), BIG2 (E-, upper panel), BIG1 (F-, G-, H-, upper panels) or GBF1 (E-, F-, G-, H-, lower panels). Bar, 10 µm.
(TIF)

Figure S3 **Coimmunoprecipitation of GBF1 and ATGL. A**- The indicated Venus-tagged GBF1 protein or region, or Venus alone, was coexpressed in Cos7 cells with HA-tagged *H. sapiens* ATGL. Immunoprecipitation was carried out with anti-GFP antibodies, and eluted proteins after immunoprecipitation were analyzed by Western blotting using anti-GFP antibody. **B**- ATGL interacts with GBF1 DCB and HUS domains. HA-tagged *H. sapiens* ATGL (full length or a deleted form) was coexpressed in Cos7 cells with the indicated Venus-tagged GBF1 domain. Immunoprecipitation was carried out with anti-GFP antibodies, and eluted proteins after immunoprecipitation were analyzed by Western blotting using anti-HA antibody. **C**- GBF1 – ATGL interactions are not affected by the S47A mutation in ATGL. HA-tagged *H. sapiens* ATGL-S47A was coexpressed in Cos7 cells with the indicated Venus-tagged GBF1 region, and co-immunoprecipitation experiments carried out as in part B. The DCB-linker constuct contains the DCB domain plus the region between DCB and HUS domains (see Figure 2).
(TIF)

Table S1 **Plasmids used in this study.**
(DOC)
