# Supplementary figures and images for: Interaction between the Triglyceride Lipase ATGL and the Arf1 Activator GBF1
Source: PLoS One. 2011 Jul 18;6(7):e21889. doi: 10.1371/journal.pone.0021889 (PMC3138737; doi:10.1371/journal.pone.0021889)

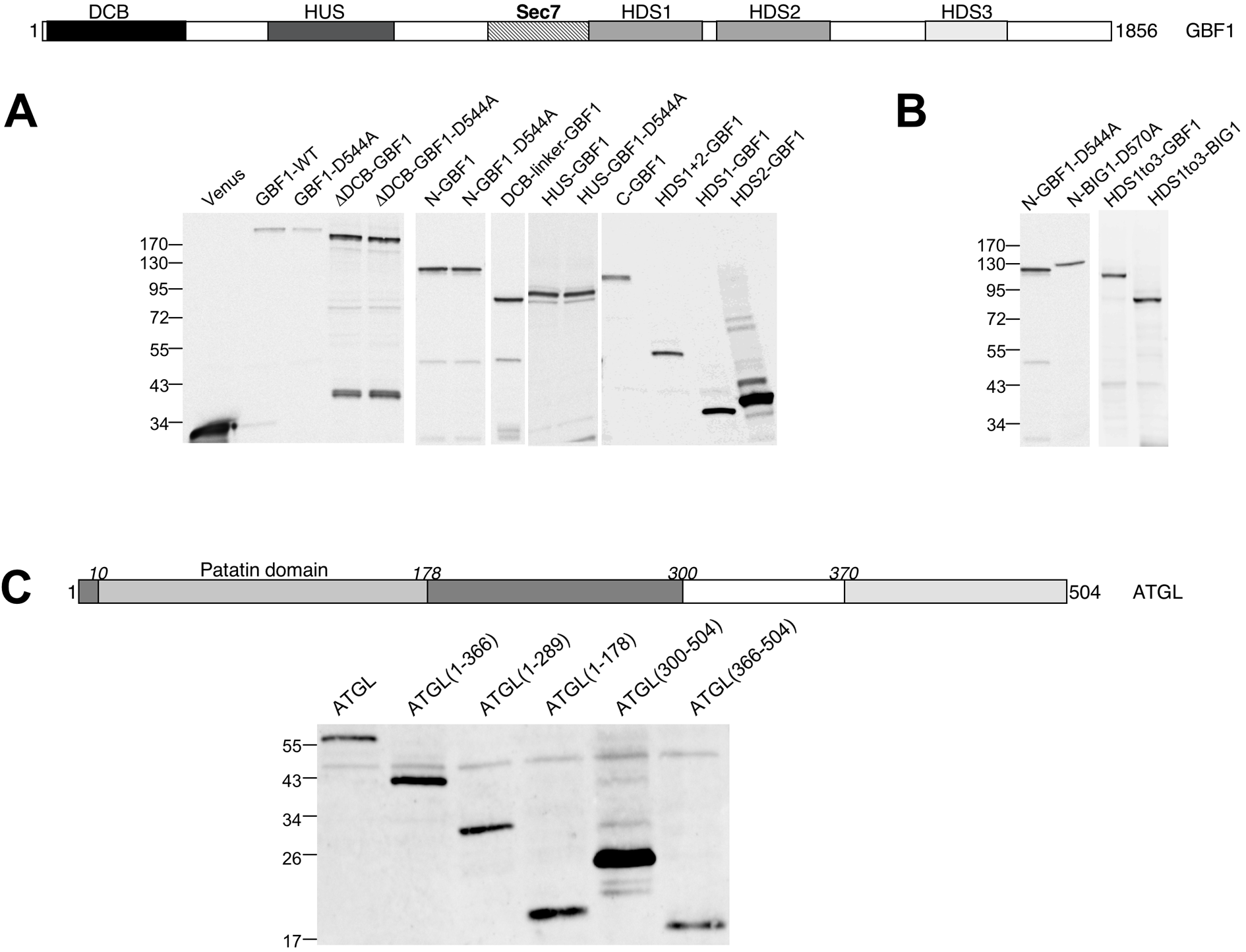

Supplement: Figure S1 — Expression level of Venus-GBF1 and HA-ATGL constructs in Cos7 cells. A- Lysates from Cos7 cells transfected with the indicated Venus-tagged GBF1 regions were analyzed by Western blotting using anti-GFP antibody. These results are representative of experiments carried out at least 3 times; co-expression of ATGL constructs did not affect GBF1 levels. The DCB-linker constuct contains the DCB domain plus the region between DCB and HUS domains (see Figure 2). B- Western blot using GFP antibodies of lysates from Cos7 cells transfected with the indicated Venus-tagged GBF1 or BIG1 construct. C- Western blot using HA antibodies of lysates from Cos7 cells transfected with the indicated HA-tagged ATGL construct. (TIF) [file pone.0021889.s001.tif]

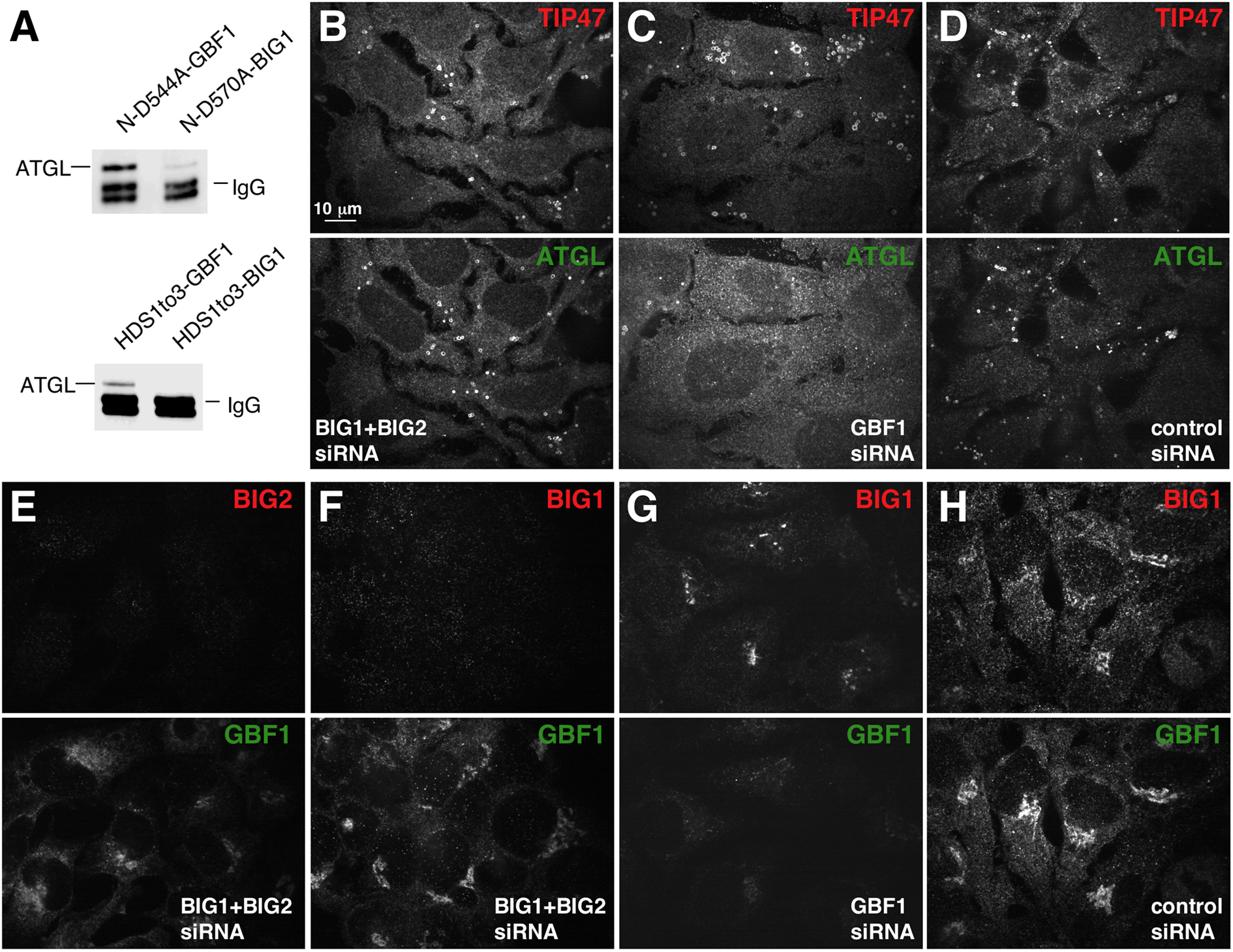

Supplement: Figure S2 — Specificity of interaction between ATGL and GBF1. A- HA-tagged H. sapiens ATGL was coexpressed in Cos7 cells with the indicated Venus-tagged GBF1 or BIG1 region. Immunoprecipitation was carried out with anti-GFP antibodies, and eluted proteins after immunoprecipitation were analyzed by Western blotting using anti-HA antibody. B- to H- Depletion of BIG1 and BIG2 does not affect ATGL association with LDs. HeLa cells were transfected with siRNAs targeting BIG1 and BIG2 together (B-, E-, F-), GBF1 (C-, G-) or lamin (negative control) (D-, H-), then immunostained with antibodies against TIP47 (B-, C-, D-, upper panels) or ATGL (B-, C-, D-, lower panels), BIG2 (E-, upper panel), BIG1 (F-, G-, H-, upper panels) or GBF1 (E-, F-, G-, H-, lower panels). Bar, 10 µm. (TIF) [file pone.0021889.s002.tif]

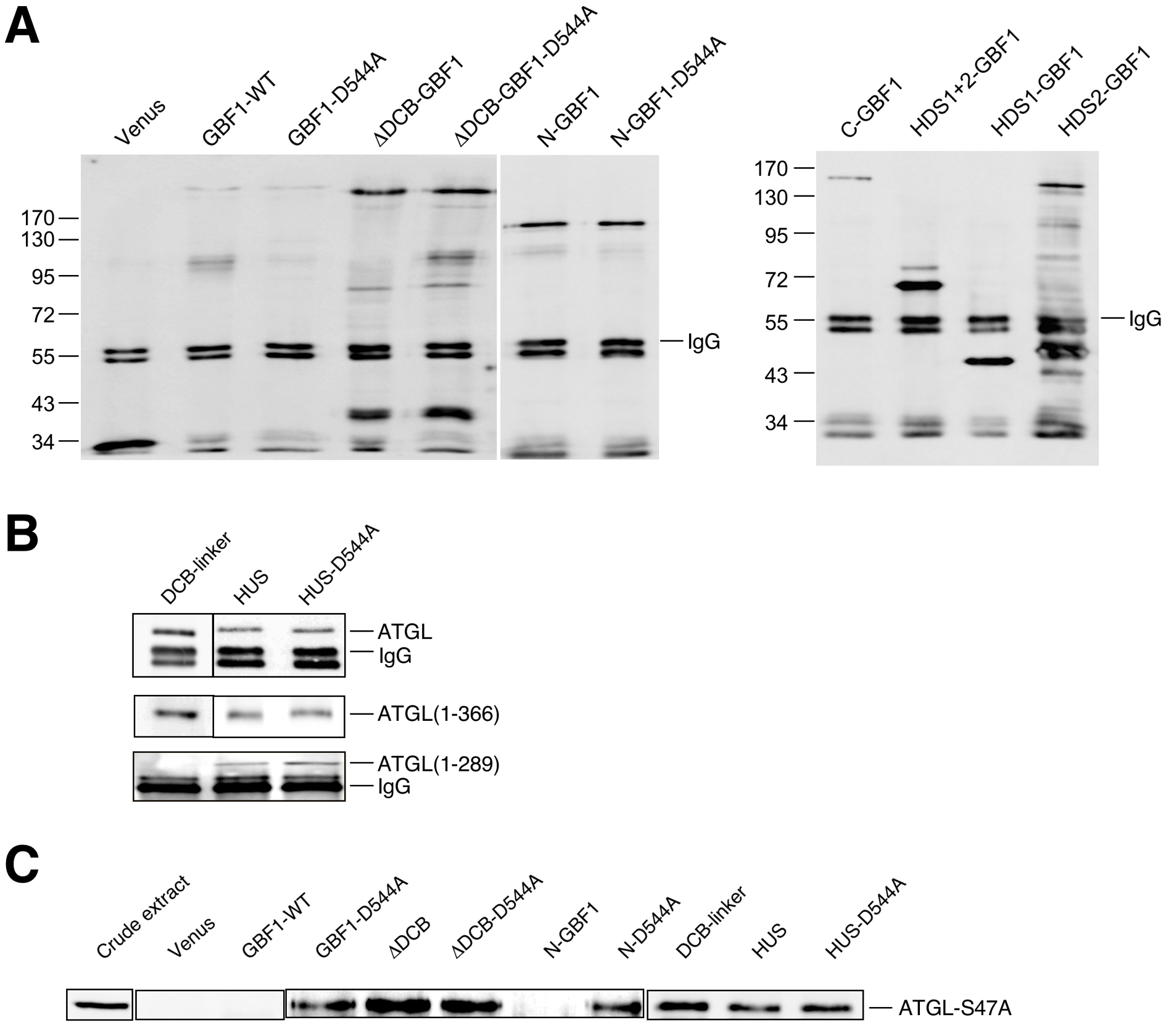

Supplement: Figure S3 — Coimmunoprecipitation of GBF1 and ATGL. A- The indicated Venus-tagged GBF1 protein or region, or Venus alone, was coexpressed in Cos7 cells with HA-tagged H. sapiens ATGL. Immunoprecipitation was carried out with anti-GFP antibodies, and eluted proteins after immunoprecipitation were analyzed by Western blotting using anti-GFP antibody. B- ATGL interacts with GBF1 DCB and HUS domains. HA-tagged H. sapiens ATGL (full length or a deleted form) was coexpressed in Cos7 cells with the indicated Venus-tagged GBF1 domain. Immunoprecipitation was carried out with anti-GFP antibodies, and eluted proteins after immunoprecipitation were analyzed by Western blotting using anti-HA antibody. C- GBF1 – ATGL interactions are not affected by the S47A mutation in ATGL. HA-tagged H. sapiens ATGL-S47A was coexpressed in Cos7 cells with the indicated Venus-tagged GBF1 region, and co-immunoprecipitation experiments carried out as in part B. The DCB-linker constuct contains the DCB domain plus the region between DCB and HUS domains (see Figure 2). (TIF) [file pone.0021889.s003.tif]
