# Supplementary material for: Interaction between the Triglyceride Lipase ATGL and the Arf1 Activator GBF1
Source: PLoS One. 2011 Jul 18;6(7):e21889. doi: 10.1371/journal.pone.0021889 (PMC3138737; doi:10.1371/journal.pone.0021889)
Supplement: Table S1 — Plasmids used in this study. (DOC) [file pone.0021889.s004.doc]

| Plasmid | Expressed protein | Reference |
| --- | --- | --- |
| pcDNA3-HA |  | Invitrogen |
| pcDNA-ATGL | HA-ATGL (*H. sapiens*) | This study |
| pcDNA-ATGL(1-366) | HA-ATGL(1-366) (*H. sapiens*) | This study |
| pcDNA-ATGL(1-289) | HA-ATGL(1-289) (*H. sapiens*) | This study |
| pcDNA-ATGL(1-178) | HA-ATGL(1-178) (*H. sapiens*) | This study |
| pcDNA-ATGL-S47A | HA-ATGL-S47A (*H. sapiens*) | This study |
| pcDNA-ATGL(1-366)-S47A | HA-ATGL(1-366)-S47A (*H. sapiens*) | This study |
| pEGFP-N1 | EGFP | Clontech |
| pEGFP-N1-ATGL(1-178) | GFP-ATGL(1-178) (*H. sapiens*) | This study |
| pEGFP-N1-ATGL(1-289) | GFP-ATGL(1-289) (*H. sapiens*) | This study |
| pEGFP-N1-ATGL(1-366) | GFP-ATGL(1-366) (*H. sapiens*) | This study |
| pEYFP-C1 | EYFP | Clontech |
| pVenus-C1 | Venus (YFP-F46L, F64L, M153T, V163A, S175G) | George Patterson |
| pTKN101 | Venus-GBF1 (*H. sapiens*) | [4] |
| pVenus-GBF1-D544A | Venus-GBF1-D544A (*H. sapiens*) | This study |
| pVenus-DCB-linker-GBF1 | Venus-GBF1(1-393) (*H. sapiens*) | This study |
| pVenus-HUS-GBF1 | Venus-GBF1(211-709) (*H. sapiens*) | This study |
| pVenus-HUS-GBF1-D544A | Venus-GBF1(211-709)-D544A (*H. sapiens*) | This study |
| pVenus-N-GBF1 | Venus-GBF1(1-709) (*H. sapiens*) | This study |
| pVenus-N-GBF1-D544A | Venus-GBF1(1-709)-D544A (*H. sapiens*) | This study |
| pVenus-C-GBF1 | Venus-GBF1(900-1856) (*H. sapiens*) | This study |
| pVenus-HDS1to3-GBF1 | Venus-GBF1(900-1641) (*H. sapiens*) | This study |
| pVenus-HDS1+2-GBF1 | Venus-GBF1(900-1278) (*H. sapiens*) | This study |
| pVenus-HDS1-GBF1 | Venus-GBF1(900-1067) (*H. sapiens*) | This study |
| pVenus-HDS2-GBF1 | Venus-GBF1(1067-1278) (*H. sapiens*) | This study |
| pEYFP-Sec7-GBF1 | YFP-GBF1(710-894) (*H. sapiens)* | [37] |
| pVenus-Sec7EK-GBF1 | Venus-GBF1(710-894)-E794K (*H. sapiens*) | This study |
| pVenus-ΔDCB-GBF1 | Venus-GBF1(298-1856) (*H. sapiens*) | This study |
| pVenus-ΔDCB-GBF1-D544A | Venus-GBF1(298-1856)-D544A (*H. sapiens*) | This study |
| pVenus-Sec7EK-BIG1 | Venus-BIG1(710-892)-E793K (*H. sapiens*) | This study |
| pVenus-Sec7EK-BIG2 | Venus-BIG2(653-840)-E738K (*H. sapiens*) | This study |
| pVenus-Sec7EK-ARNO | Venus-ARNO(72-253)-E156K (*H. sapiens*) | This study |
| pVenus-Sec7EK-EFA6 | Venus-EFA6(521-716)-E621K (*H. sapiens*) | This study |
| pVenus-N-BIG1-D570A | Venus-BIG1(1-709)-D570A (*H. sapiens*) | This study |
| pVenus-HDS1to3-BIG1 | Venus-BIG1(893-1489) (*H. sapiens)* | This study |
| pGEX-4T1 |  | GE Healthcare |
| pGEX-4T1-ATGL(1-366) | GST-ATGL(1-366) (*H. sapiens*) | This study |
| pGEX-4T1-ATGL(300-504) | GST-ATGL(300-504) (*H. sapiens*) | This study |
| pGEX-4T1-ATGL(1-178) | GST-ATGL(1-178) (*H. sapiens*) | This study |
| pBAD-HISC |  | Invitrogen |
| pBAD-Sec7-GBF1 | His-GBF1(710-894) (*H. sapiens*) | This study |
| pBAD-DCB-GBF1 | His-GBF1(1-210) (*H. sapiens)* | This study |
| pBAD-HUS-GBF1 | His-GBF1(211-709) (*H. sapiens*) | This study |
| pET22b |  | Novagen |
| pET22b-HDS1-GBF1 | GBF1(908-1065)-His (*H. sapiens*) | This study |
| pGBKT7 |  | Clontech |
| pGBKT7-Sec7-GBF1 | DNA-BD-GBF1(711-895) (*H. sapiens*) | This study |
| pGBKT7-Sec7-BIG2 | DNA-BD-BIG2(654-840) (*H. sapiens*) | This study |
| pGBKT7-Sec7-EFA6 | DNA-BD-EFA6 (147-336) (*H. sapiens*) | This study |
| pGBKT7-Sec7-ARNO | DNA-BD-ARNO (72-252) (*H. sapiens*) | This study |
| pGBKT7-ATGL(300-370) | DNA-BD-ATGL(300-370) (*H. sapiens*) | This study |
| pGBKT7-ATGL(300-504) | DNA-BD-ATGL(300-504) (*H. sapiens*) | This study |
| pGBKT7-ATGL(366-504) | DNA-BD-ATGL(366-504) (*H. sapiens*) | This study |
| pGADT7 |  | Clontech |
| pGADT7-Sec7-GBF1 | AD-GBF1(711-895) (*H. sapiens*) | This study |
| pGADT7-DCB-GBF1 | AD-GBF1(2-202) (*H. sapiens*) | This study |
| pACT2-ATGL(366-504) | AD-ATGL(366-504) (*H. sapiens*) | This study |
